# Analysis of random PCR‐originated mutants of the yeast Ste2 and Ste3 receptors

**DOI:** 10.1002/mbo3.361

**Published:** 2016-05-05

**Authors:** Serena Gastaldi, Michela Zamboni, Giulia Bolasco, Gianfranco Di Segni, Glauco P. Tocchini‐Valentini

**Affiliations:** ^1^ CNR, Institute of Cell Biology and Neurobiology (IBCN)Monterotondo (Rome)00015Italy; ^2^ EMBL, European Molecular Biology LaboratoryMonterotondo (Rome)00015Italy

**Keywords:** G protein‐coupled receptors, hyperactive mutations, mating response, signal transduction

## Abstract

The G protein‐coupled receptors Ste2 and Ste3 bind α‐ and **a**‐factor, respectively, in *Saccharomyces cerevisiae*. These receptors share a similar conformation, with seven transmembrane segments, three intracellular loops, a C‐terminus tail, and three extracellular loops. However, the amino acid sequences of these two receptors bear no resemblance to each other. Coincidently the two ligands, α‐ and **a**‐factor, have different sequences. Both receptors activate the same G protein. To identify amino acid residues that are important for signal transduction, the *STE2* and *STE3* genes were mutagenized by a random PCR‐based method. Mutant receptors were analyzed in *MAT*α cells mutated in the *ITC1* gene, whose product represses transcription of **a**‐specific genes in *MATα*. Expression of *STE2* or *STE3* in these cells results in autocrine activation of the mating pathway, since this strain produces the Ste2 receptor in addition to its specific ligand, α‐factor. It also produces **a**‐factor in addition to its specific receptor, Ste3. Therefore, this strain provides a convenient model to analyze mutants of both receptors in the same background. Many hyperactive mutations were found in *STE3*, whereas none was detected in *STE2*. This result is consistent with the different strategies that the two genes have adopted to be expressed.

## Introduction

G protein‐coupled receptors (GPCRs) constitute the major class of signal transducing proteins, present in all eukaryotic organisms from yeasts to mammals. Several hundred different GPCRs are encoded by the human genome, responding to a variety of stimuli, such as hormones, neurotransmitters, odorants, and light signals (reviewed in Latek et al. 2012; Alexander et al. 2013). GPCRs are also the most common target for pharmaceutical drugs (Salon et al. 2011; Granier and Kobilka 2012). Although their sequences may be completely unrelated to each other, they are all predicted to share a common molecular structure, consisting of seven transmembrane hydrophobic helices linked by alternating intracellular and extracellular loops, an N‐terminal extracellular chain and usually a long cytoplasmic C‐terminal tail (Strader et al. 1994; Kakarala and Jamil 2014).

Receptors activated by their specific ligands transmit the signal to several proteins, especially to the heterotrimeric G proteins, promoting the exchange of GDP with GTP on the Gα subunit (Conklin and Bourne 1993; McCudden et al. 2005; Wettschureck and Offermanns 2005; Oldham and Hamm 2008). The binding of GTP causes the dissociation of Gα from G*βγ*, leading to the specific regulation of target effectors. Upon GTP hydrolysis, the α and *β*γ subunits reassociate and the G protein returns to the receptor in its resting state (Malbon 2005; Dupré et al. 2009; Khan et al. 2013).

The yeast *Saccharomyces cerevisiae* is a useful organism for studying signal transduction pathways involving GPCRs (Sprague and Thorner 1992; Bardwell 2005; Dohlman and Slessareva 2006; Ydenberg and Rose 2008). Each of the two haploid mating cell types (*MAT* **a** and *MAT*α) secrete a peptide pheromone (**a**‐factor and α‐factor, respectively) that acts on the other cell type to promote conjugation, resulting in the formation of *MAT* **a**/α diploid cells. The **a**‐ and α‐mating factors bind to cell surface GPCRs, encoded, respectively, by the *STE3* and *STE2* genes. In response to pheromone binding the receptors, the G protein α subunit replaces bound GDP with GTP and dissociates from the G protein *βγ* complex, which in turn triggers a cascade of events leading to transcriptional induction of specific genes, stimulation of morphological changes, and inhibition of growth. The deletion of the gene encoding the yeast Gα subunit (*GPA1*) results in lethality in haploid cells because the free G*βγ* complex constitutively activates the pathway leading to growth inhibition. The Ste2 and Ste3 receptors share a similar general conformation, common to all GPCRs, but their amino acid sequences bear no resemblance to each other. This is consistent with the fact that the two pheromone peptides, **a**‐ and α‐factor, have different sequences. In contrast, both receptors activate the same heterotrimeric G protein.

Several studies were performed to characterize the yeast receptors through the identification of mutations affecting their function. The Ste2 receptor was highly studied (see, among the others, Konopka et al. 1996; Sommers and Dumont 1997; Dube and Konopka 1998; Sommers et al. 2000; Martin et al. 2002; Celic et al. 2003; Mathew et al. 2011; Zuber et al. 2015). On the other hand, the Ste3 receptor was analyzed to a lesser extent (Mackay and Manney 1974; Hagen et al. 1986; Boone et al. 1993). In particular, the role of the third intracellular loop of the Ste3 receptor was investigated in the past by a series of mutations and deletions, and it was found that this region may have a negative regulatory effect, maintaining the receptor in an inactive state until stimulation by the ligand occurs (Boone et al. 1993). Mutational analysis of the corresponding region in the Ste2 receptor showed that this loop is crucial for the activation of the G protein, as well as ligand discrimination and receptor internalization (Weiner et al. 1993; Clark et al. 1994; Stefan and Blumer 1994; Stefan et al. 1998).

In order to identify amino acid residues that are important for signal transduction in different regions of the receptors, we performed a comparative analysis of mutations of the *STE2* and *STE3* genes. Large portions of *STE2* and *STE3* were mutagenized by a random PCR‐based method (Dosil and Konopka 2010). Mutant receptors were analyzed in a strain that we recently found, which has the remarkable feature of being able to express both the Ste2 and Ste3 receptors and activate them in an autocrine way (Di Segni et al. 2011). This strain is therefore a very convenient model to analyze, in the same genetic background, a large number of mutations in two different receptors, which interact with the same G protein to activate the same pathway.

## Experimental Procedures

### Strains

*Escherichia coli* strain DH5‐α was used to propagate plasmid DNA. *S. cerevisiae* strains were as follows: DDS2 (*MAT*α, *ste2Δ*,* ste3Δ::ADE2*,* far1Δ, fus1*::*FUS1‐lacZ‐TRP1*,* sst2*,* ura3‐52*,* leu2‐3*,* trp1*,* ade2‐1*,* lys2‐1*,* his3*) derived from strain RM27 (Di Segni et al. 2008); DDS4 (*MAT*α, *ste2Δ*,* ste3Δ::ADE2*,* far1Δ, fus1*::*FUS1‐lacZ‐TRP1*,* ura3–52*,* leu2–3*,* trp1*,* ade2–1*,* lys2–1*,* his3*) derived from strain RM27; M18 (*MAT*α, *ste2Δ*,* ste3Δ::ADE2*,* far1Δ, fus1*::*FUS1‐lacZ‐TRP1*,* sst2*,* ura3–52*,* leu2–3*,* trp1*,* ade2–1*,* lys2–1*,* his3, itc1*) derived from strain DDS4 (Di Segni et al. 2011); RM6 (*MAT**a***,* ste2Δ, ade2, his3, leu2‐3, ura3‐52, trp1, fus1*::*FUS1‐lacZ‐TRP1*) (Medici et al. 1997).

### Plasmids

The *STE2* gene is derived from a 4.3‐kb *Bam*HI‐*Bam*HI fragment contained in a YEp24 plasmid, a gift from L. Hartwell (Konopka et al. 1988). The HA‐epitope for western analysis was synthesized with oligonucleotides P1 and P2 (see Table S3) and fused in frame at the 3′ end of the coding region of *STE2*;* STE2‐*HA was then cloned in pYX123, a yeast shuttle vector (R&D Systems) containing the *CEN* sequence, the *HIS3* gene, and the *GAL* promoter. A unique *Mlu*I site in the 5′‐UTR of *STE2* was used to create pYX123‐*STE2*‐HA. Plasmid p*STE2*‐Δ297 was derived from the latter and contains the *STE2* coding region without the C‐terminal region from position 297 onward. Plasmid pSM1 is derived from pYX123‐*STE2*‐HA where the coding region of *STE2*‐HA is placed under the control of the *TPI* promoter instead of the *GAL* promoter. Plasmid pSM2‐Δ307 contains the *STE3* gene and was constructed by replacing the *STE2* sequence of pSM1 with the *STE3*‐HA sequence without the C‐terminal region from position 307 onward, obtained by PCR using primers P3 and P4 and yeast genomic DNA as the template. Plasmid pSM3 bears the full‐length coding region of *STE3*‐HA. We used a modified version of *STE3* where a *Mlu*I restriction site was created by mutating the ACGAGA sequence into ACGCGT, changing A184 into C and A186 into T avoiding changes in the protein sequence.

### Oligodeoxyribonucleotides

The primers used for PCR, plasmid constructs, and sequencing are listed in Table S3.

### Mutagenesis of *STE2* and *STE3*

To generate a large collection of receptor mutants, plasmids containing portions of *STE2* or *STE3* (as detailed in the Results) were randomly mutagenized by performing PCR under error‐prone conditions using the Genemorph II Random Mutagenesis Kit (Stratagene, La Jolla, CA) according to the manufacturer's instruction. Plasmids p*STE2*‐Δ297 and pSM2‐Δ307 were used as templates. Primers pair 5 and 6 for *STE2* and primers 3 and 7 for *STE3* were used. Mutagenized PCR fragments were then cotransformed into yeast strain M18 (*MAT*α, *ste2*Δ, *ste3*,* itc1*) together with plasmid p*STE2*‐Δ297 or pSM2‐Δ307 that were linearized at a single site by digestion, respectively, with *Cla*I or *Pme*I. Growth on medium lacking histidine selected for cells carrying circular plasmids incorporated a mutagenized PCR fragment via homologous recombination.

### Transferring of mutations into full‐length *STE2* or *STE3*

Mutated plasmids were used as a template for PCR amplifications using *Pfu* DNA‐polymerase (Stratagene) or Accuprime *Taq* DNA Polymerase System (Invitrogen, Life Technology, Carlsbad, CA, USA). In the *STE2* subcloning, if the mutation was positioned at the 5′ of the *Cla*I site, the mutant plasmid was amplified with primers 8 and 6. The PCR product was cut with *Eco*RI and *Cla*I, and cloned into pSM1 cut with the same enzymes. On the other hand, if the mutation was placed at the 3′ of the *Cla*I site, the mutated plasmid was amplified with primers 9 and 10. The PCR product was cut with *Cla*I and *Sac*I and cloned into pSM1 cut in the same way. In some cases, two PCRs were done followed by a third one. The product was then cut with *Eco*RI and *Cla*I or *Eco*RI and *Sac*I, and inserted into pSM1 cut in the same way (see Table S1).

In the case of *STE3*, if the mutation was placed at the 5′ of the *Pme*I site, the mutant plasmid was amplified with primers 11 and 12. The PCR product was cut with *Mlu*I and *Pme*I and cloned into pSM3 cut in the same way. If the mutation was positioned at the 3′ of the *Pme*I site, specific sets of primers were used (Table S1). The products containing the mutation were then digested with *Mlu*I and *Sal*I, and cloned into pSM3 digested the same way.

To separate some multiple mutations of *STE2* and *STE3*, PCRs were done using specific set of primers carrying the mutation on plasmids pSM1 or pSM3 (Table S1).

### DNA sequencing

Plasmids from colonies were rescued by the rapid isolation method (Treco and Lundblad 1994) and subjected to DNA sequence analysis using a Big Dye Sequencing Kit (Applied Biosystems; Invitrogen Life Technology, Foster City, CA, USA) with appropriate primers. Sequences were analyzed on an ABI Prism 3100 Avant Genetic Analyzer (Applied Biosystems; Invitrogen Life Technology).

### Quantitative *β*‐galactosidase assay

*STE2* and *STE3* mutants were quantitatively assayed for the ability to induce *FUS1*‐*lacZ* expression. Cells from a mid‐log phase culture were diluted to A_600 _= 0.2 and incubated for 4 h (for *STE2*) or 3 h (for *STE3*) at 30°C without an external source of mating factors in the case of autocrine activation, or using a cell‐free broth of an overnight culture of cells of the opposite mating type when activation must be induced exogenously. Cells were then harvested and suspended in 1 mL of Z‐buffer (60 mmol/L Na_2_HPO_4_, 40 mmol/L NaH_2_PO_4_, 10 mmol/L KCl, 1 mmol/L MgSO_4_, 50 mmol/L *β*‐mercaptoethanol, pH 7), and two drops of chloroform were added. The cell suspension was incubated at 30°C for 15 min, and then 200 *μ*L (4 mg/mL) of chlorophenol‐red‐*β*‐D‐galactopyranoside (CPRG, Boehringer, Ingelheim am Rhein, Germany) or o‐nitrophenyl *β*‐D‐galactopyranoside (ONPG, Sigma, Saint Louis, MO, USA) were added as substrate. At appropriate times, the absorbance was measured, respectively, at 574 nm or 420 nm, and the relative *β*‐galactosidase activity was calculated with respect to wild‐type receptors.

### Western blot analysis

Cells from an overnight culture were harvested and lysed by agitation with glass beads in 200 *μ*L of buffer containing 200 mmol/L Tris‐HCl (pH 8), 1 mmol/L EDTA, 150 mmol/L (NH_4_)_2_ SO_4_, 10% glycerol, 2 mmol/L DTT, and Protease Inhibitor Complete 1X (Roche Diagnostics, Mannheim, Germany). The lysate was cleared twice by centrifugation. Protein concentration was measured by the dye‐binding assay using protein assay reagent (Bradford Protein Assay, Bio‐Rad, Hercules, CA, USA) according to the manufacturer's instructions. A total of 20 or 30 *μ*g of protein were loaded onto SDS/PAGE gels. After transfer, the membrane was probed with rat monoclonal horseradish peroxidase‐conjugated anti‐HA antibodies (high affinity 3F10, Roche, Basilea, Switzerland). The blots were developed using the ECL western blotting detection reagents (Amersham Biosciences). Bio‐Rad Precision Plus Protein WesternC Standards were used as markers.

### GFP receptor fusion proteins

To localize the receptors in living cells, we constructed plasmids which express the green fluorescent protein (GFP) fused to receptors Ste2 or Ste3 at their C‐termini. Plasmid pSM1‐*STE2*‐GFP was generated with primers P50 and P51 and pGFP(S65T)‐CAM plasmid (a gift from G. Papoff) as a template. The PCR product was digested by *Hind*III and *Xho*I, and ligated to full‐length *STE2* cut using the same enzymes. Plasmid pSM3‐*STE3*‐GFP was obtained with a three‐step ligation. A PCR reaction with primers P52 and P51 generated GFP(S65T) coding sequences flanked by *Xho*I and *Pst*I restriction sites. The PCR product was digested with *Xho*I and *Pst*I, and ligated together with a PCR product obtained from pSM3 with primers P10 and P11, after digestion with *Pst*I and *Mlu*I. The ligated product was cloned into pSM3 previously excised with *Xho*I and *Mlu*I.

### Confocal laser scanning microscopy

Samples (5 mL) were grown over night up A_600_ = 1 × 10^7^, resuspended in 1 mL of broth, and then incubated for 4 h at 30°C. To label acidic organelles such as vacuoles, samples were resuspended in 250 *μ*L 10 mmol/L HEPES buffer pH 7.4 containing 5% glucose with 100 *μ*mol/L Cell Tracker Blue CMAC (Life Technologies), and incubated for 30 min at room temperature. Samples were spun for 3 min at 6700 g and the pellet resuspended in 1 mL of low melting agarose (Bio‐Rad) dissolved in the HEPES buffer. Aliquotes of 10 *μ*L for each sample were seeded on glass bottom dishes (35 mm ø, ibidi) and covered with a 13 mm ø glass cover slip. Cells were observed with a laser scanning confocal microscope, TCS SP5 (Leica Microsystems, Mannheim) using a Plan Apo 63×(NA = 1.20) water‐immersion lens with optical pinhole at 1 AU. An Argon multiline laser operating at 488 nm (GFP), and a UV Diode laser at 405 nm (blue vacuole CMAC marker) were used as excitation sources. Confocal Z‐stacks were collected with 0.29 mm interval in a 5 mm total optical depth. Images for direct comparison were collected under the same parameters and representative images were chosen among 20 cells in multiple assays.

## Results

The mutagenesis of *STE2* and *STE3* was performed on 3′‐end‐truncated genes, given that pheromone receptors devoid of the C‐terminal region elicit a more elevated response in signal transduction (Konopka et al. 1988; Reneke et al. 1988). The higher the response to pheromones, the easier the detection of mutations affecting signal transduction will be. The supersensitive phenotype of the truncated receptors is caused by the inability of the Sst2 protein, a regulator of G‐protein signaling (RGS), to bind the C‐terminus of the receptor and therefore to inhibit its response (Dohlman et al. 1996; Apanovitch et al. 1998; Ballon et al. 2006). Moreover, the C‐terminal region of the receptor is phosphorylated upon activation by pheromone binding, which is the prelude to ubiquitination and clathrin‐mediated endocytosis: the absence of endocytosis allows, as a consequence, higher sensitivity (Tan et al. 1993; Chen and Konopka 1996; Roth and Davis 1996; Hicke et al. 1998; Alvaro et al. 2014).

The mutagenesis was carried out on a substantial part of the receptors, in particular, the region encompassing four transmembrane segments (for Ste2) or five (for Ste3) and their connecting loops, a region which according to much previous work is the most important for signal transduction. Random mutagenesis was accomplished using PCR under error‐prone conditions. PCR fragments were then mixed with a *HIS3*‐bearing centromere‐plasmid that contained the tail‐truncated receptor gene, linearized at a single site. Since linear plasmids cannot replicate inside yeast cells, plating cells transformed with this DNA mixture onto histidine‐minus plates selected for circular plasmid molecules derived by homologous recombination between linear plasmids and PCR fragments. We used the *MAT*α yeast strain M18, which bears several traits particularly useful for the screening of mutant receptors (Di Segni et al. 2011): both the endogenous *STE2* and *STE3* genes are deleted, to avoid interference with mutant genes. The growth‐arrest *FAR1* gene, which is normally expressed upon pheromone binding, is deleted as well, so that activated cells can grow and form colonies. The M18 strain also bears a null mutation in *HIS3*, necessary for the selection of the recombinant plasmids, and carries the highly pheromone‐responsive *FUS1*‐*lacZ* reporter gene, which produces a blue color on plates containing 5‐bromo‐4‐chloro‐3‐indolyl *β*‐D‐galactopyranoside (X‐Gal). This strain is also mutated in the *SST2* gene, whose mutants have a supersensitive phenotype.

**Figure 1:**
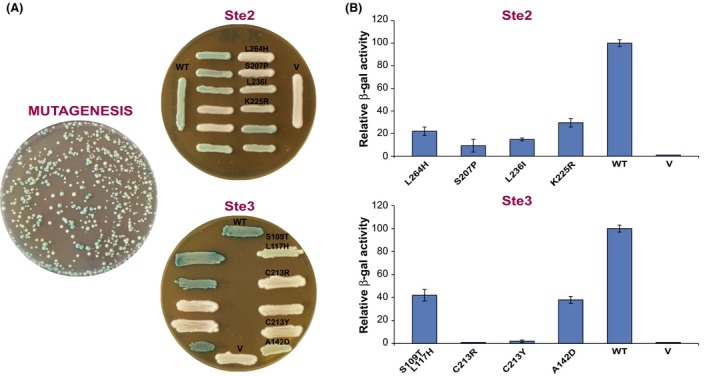
Identification of Ste2 and Ste3 receptor mutants with reduced mating activity. (A) The autocrine M18 strain, harboring the *STE2* or *STE3* genes mutagenized by a random PCR‐based method, was plated on an X‐Gal medium (the plate shown is for *STE2*; similar plates were obtained for *STE3*). Colonies appearing white or light‐blue, as opposed to the deep blue of colonies containing nonmutagenized genes, were picked up and patched again to confirm the colony phenotype. (B) and (C) Quantitative *β*‐Gal assay of the indicated white or light‐blue mutants of, respectively, the Ste2 and Ste3 receptors. The results for each mutant represent the averages of three samples, normalized to the level of *β*‐Gal activity of cells expressing the nonmutagenized receptors; error bars correspond to 1 standard deviation (SD).

The most important feature of the M18 strain is that it is also mutated in *ITC1*, a gene whose product together with the product of *ISW2*, is known to repress transcriptional initiation of *STE2* and other **a**‐specific genes in *MATα* cells, such as the **a**‐factor gene and the *BAR1* protease (Ruiz et al. 2003). The *itc1* mutation confers a notable property regarding this study because it renders the M18 strain autocrine for both receptors, Ste2 and Ste3. In fact, the *STE2* gene, although being an **a**‐specific gene, is expressed in M18, leading to autocrine activation by the α‐factor that this *MAT*α strain naturally produces. On the other hand, the production of **a**‐factor, achieved by the derepression of its **a**‐specific coding gene, leads to the autocrine activation of the Ste3 receptor normally expressed in this strain. This characteristic of M18, therefore, makes it possible to analyze and compare mutations in both *STE2* and *STE3* in the same strain (Fig. 1A).

The autocrine property of M18 means that there is no need to provide any kind of pheromone in the culture medium because the cell itself produces both the receptor and the ligand necessary to activate the pathway and consequently, the *FUS1*‐*lacZ* reporter gene. This fact permits the identification of mutants just by observing the blue color phenotype. Colonies can be produced due to the deletion of the growth‐arrest *FAR* gene. Thousands of colonies could therefore be screened easily and rapidly with this method.

### *STE2* mutants

**Figure 2:**
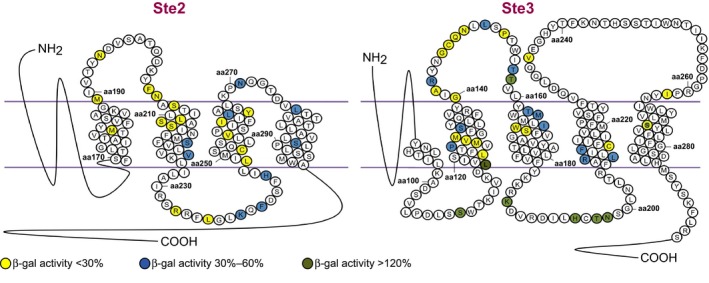
Distribution of the mutations in the Ste2 or Ste3 receptors. The predicted topological structures of the seven transmembrane segments of the Ste2 and Ste3 receptors are shown. Only the amino acids subjected to random mutagenesis are depicted as circles. The relative *β*‐Gal activity induced by the mutated full‐length receptors was determined in strain RM6 (for Ste2) or DDS4 (for Ste3). Mutated residues are colored as indicated.

The C‐terminally truncated *STE2* gene was missing the entire coding region of the tail, from codon 296 through its end. The portion of the *STE2* gene subjected to random PCR‐mutagenesis encompassed the region encoding transmembrane segments 4 through 7, together with the second and the third extracellular loop as well as the third intracellular loop (Fig. 2). PCR fragments were then transformed together with a *HIS3*‐bearing centromere‐plasmid containing the tail‐truncated receptor gene with its endogenous promoter, linearized by digestion with *Cla*I at a single site in the middle of the gene. Two different mutagenesis experiments were performed. Twenty plates like the one shown in Figure 1A were screened, for a total of about 4000 colonies: 108 colonies displayed a white or light‐blue color, indicating total or partial inability to perform as receptors. Candidate mutants were streaked on a fresh plate and plasmids were rescued from colonies for further analysis.

The *STE2* sequence of candidate mutants was analyzed and compared with the wild‐type gene. In 14 candidates, the sequence was identical to wild type and they were discarded: presumably, the mutation occurred in another gene involved in the mating response whose effect is to inhibit the transduction pathway. In 12 cases, a deletion of one or more nucleotides of *STE2* arose, causing a frameshift mutation, while in 14 mutants, a stop codon was produced. These colonies were discarded as well, being noninformative.

Missense mutations were found in 68 cases, so distributed: 36 single mutants, 20 double mutants, nine candidates with three mutations, and three with more than three mutated codons. In several cases, the same mutation was obtained more than once: in particular, L264H occurred four times, F241S twice, L236I/H/P twice each, S214P twice, S207P three times.

We continued the analysis on 56 mutants bearing a single or a double mutation. To confirm that the mutant phenotype was plasmid dependent, the rescued plasmids were retransformed into the M18 strain and analyzed with a liquid, quantitative *β*‐galactosidase (*β*‐Gal) assay. Figure 1B represents the results of some typical mutants. Thirty of them displayed an activity lower than 60% of that of the wild‐type Ste2 receptor; they were further characterized, choosing this level as a cutoff for defining dysfunctional signal transduction. We then transferred the DNA region containing the mutation into the full‐length receptor gene by PCR methods, in order to reconstitute the original gene context. Next, we inserted the complete gene into the plasmid pSM1, which contains the HA‐epitope coding region at the 3′‐end. It also bears the strong and constitutive promoter of the *TPI* gene, encoding triose phosphate isomerase (Schiestl and Gietz 1989): this promoter avoids the dependence of transcription activation on the mating type. Double mutations were separated from each other (see Table S1). Candidate mutants were first analyzed in the strain RM6, which is neither autocrine nor supersensitive and, therefore, more similar to physiological conditions. Receptor activation in this nonautocrine strain was achieved using a cell‐free broth of a culture of *MAT*α cells as α‐factor source (strain GDS30). We used this source of α‐factor instead of the commercial one in order to compare the results to the analysis of the Ste3 receptor, for which there is no commercial **a**‐factor. In order to standardize the experimental conditions, *MAT*α cells used as α‐factor source were at the same level of time incubation and optical density in each experiment, and we repeated the assays several times. Moreover, in each assay, we compared the mutant receptors against the wild‐type, as an internal standard.

Twenty‐seven mutants showed activity lower than 60% of that of the wild‐type Ste2 receptor (Fig. 2 and Table S2) and were further characterized with different strains.

**Figure 3:**
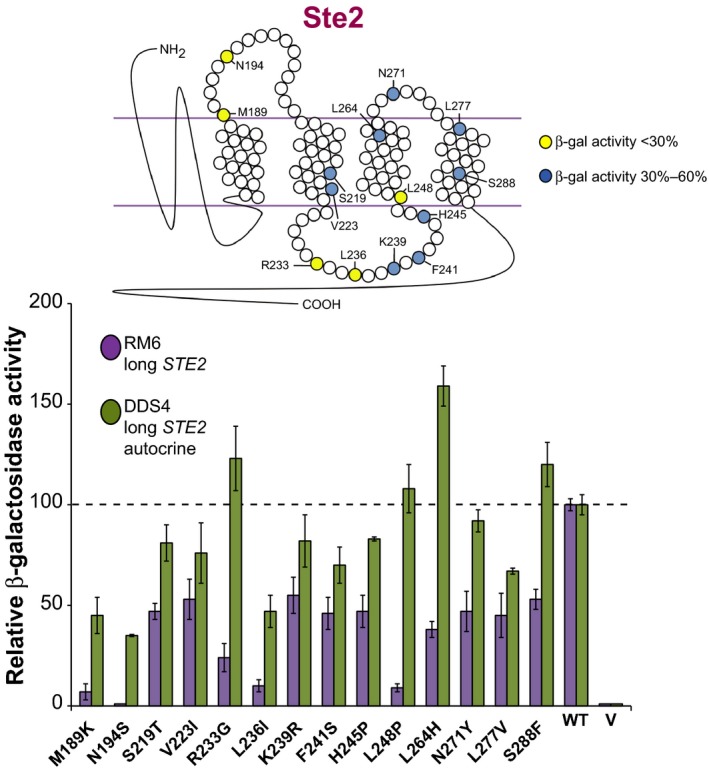
Mutants of the Ste2 receptor with lower activity in a nonautocrine strain. The position of mutated residues is depicted on the top. Plasmids coding for the indicated mutant or wild‐type Ste2 receptors were introduced into two different strains, the nonautocrine RM6 (*MAT* **a)** and the autocrine DDS4 (*MAT*α strain). RM6 cells were incubated for 4 h together with a cell‐free broth of an overnight culture of *MAT*α strain GDS30, as a source of α‐factor. Autocrine DDS4 cells were incubated for 4 h in the absence of exogenous α‐factor. The results for each mutant represent the average of three samples, normalized to the level of *β*‐Gal activity of cells expressing the nonmutagenized receptors; error bars correspond to 1 SD. The horizontal line shows the value for wild‐type receptor.

We assayed these mutants in an autocrine but not supersensitive strain (DDS4), comparing the results to those obtained with RM6, which is neither autocrine nor supersensitive. For 14 amino acid residues, we found that in the autocrine strain, the activity of the mutants was increased with respect to the nonautocrine strain (Fig. 3).

Regarding mutations in residues M189K, N194S, R233G, L236I, L248P, L264H, which represent every domain of the Ste2 receptor such as the transmembrane segments and the cytoplasmic or extracellular loops, enhancement of the *β*‐Gal activity was at least 4–5 times. This increase cannot be solely attributed to different extents of growth between *FAR1* ^+^ RM6 cells, which are inhibited in responsive conditions, and the autocrine, *far1*‐deleted DDS4 cells, which are not. A likely explanation of the increase in the response in an autocrine strain with respect to nonautocrine cells is that the defect in these mutants causes a lower affinity to the pheromone (see in the Discussion). This low affinity, therefore, might be relieved in the autocrine strain if the local concentration of the pheromone is higher in the vicinity of the receptor, due to the receptor being physically close to the area where the pheromone is secreted (Brizzio et al. 1996).

**Figure 4:**
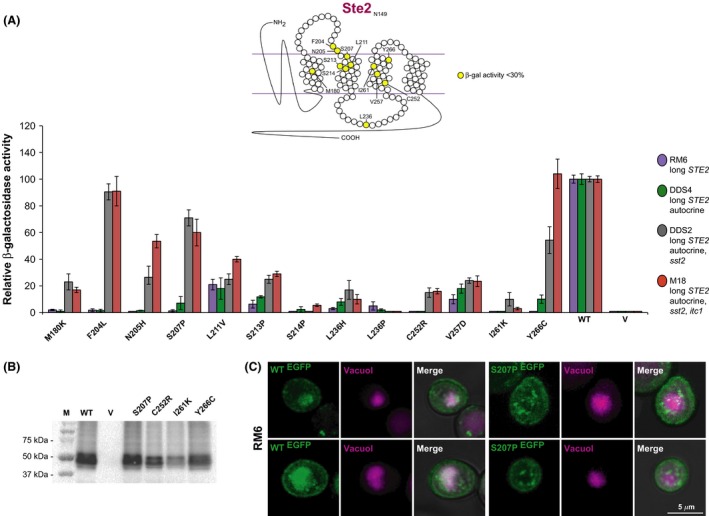
Analysis of selected down mutations of the Ste2 receptor in different strains. (A) The position of mutated residues of the Ste2 receptor is depicted on the top. The *β*‐Gal activity of different strains carrying plasmids coding for the indicated mutants is shown. Data are normalized with respect to wild type. The main features of the strains are indicated. The *sst2* mutation confers supersensitivity; the *itc1* mutation permits the expression of the *BAR1* protease, therefore decreasing the amount of α‐factor. (B) Western blot. The RM6 strain was transformed with plasmids coding for the indicated null mutant or wild‐type (WT) receptors, or with the empty vector (V). Total cell lysates (30 *μ*g each sample) were loaded on polyacrylamide gels and analyzed by western blot using anti‐HA antibodies. Protein standards are shown in lane M. (C) Confocal microscopy analysis on Ste2‐GFP (WT) and mutant Ste2‐S207P‐GFP in RM6 strain, two samples each. Wild‐type Ste2‐GFP receptor (*green*) appears to be localized at the cell membrane and in vesicles around the vacuolar lumen (*magenta*). In S207P mutant, GFP signal (*green*) seems localized at the perivacuolar area (*magenta*), whereas it appears less intense at the cell membrane. Not in all cells the vacuole was visible. GFP, green fluorescent protein.

For the other 13 mutations, the autocrine feature alone did not rescue the *β*‐Gal activity, and only the concomitance of supersensitivity conferred by the *sst2* mutation increased the response in some cases (Fig. 4A, especially mutants F204L, N205H, S207P, Y266C). In a couple of these mutants (N205H and Y266C), the *itc1* mutation in the M18 strain further increased the response. In other cases (S214P, L236P, I261K), the amino acid change produced a null mutation in all strains we checked.

We verified the presence of the Ste2 protein in the null mutants in RM6 by Western analysis using anti‐HA antibodies. The protein is indeed abundant in null mutants S207P and Y266C, and at a lower level in C252R and I261K (Fig. 4B). We also checked the expression of other mutants by Western analysis. In most of them, there was no significant decrease in the amount of receptor expression. In some, specifically M180K, S213P, S214P, R233G, L236I and L236P (but not L236H), H245P, L264H, there was a decrease in the amount of the Ste2 receptor, such as for I261K (Fig. S1).

In order to visualize the receptors in yeast cells, we linked the 3′‐end of *STE2* to the gene encoding the GFP. Although it is possible that such a fusion is not properly folded, we determined that there is no difference in the response between receptors with or without the GFP (Fig. S2). Therefore, valuable indications may be obtained by these constructs concerning the localization of the different mutant receptors (Stefan and Blumer 1999; Alvaro et al. 2014). When yeast cells are observed by confocal microscopy, the wild‐type Ste2‐GFP receptor appears to be localized at the cell membrane as well as within the vacuole in strain RM6. Instead, the mutant Ste2‐S207P, which is null in RM6, appears to be mainly localized at the perivacuolar area in discrete sites, while it is less present at the cell membrane (Fig. 4C). For any of the autocrine strains tested, such as DDS4, DDS2, and M18, the GFP signal seems diffused throughout the cytoplasm for the wild‐type receptor, whereas for the mutant, the GFP signal appears to be localized around the vacuole as in RM6 (Fig. S3). This result might be explained by the higher recycling rate of the wild‐type receptor in the autocrine and/or supersensitive strains, with respect to the null mutant.

### *STE3* mutants

The Ste3 receptor used in the present work for mutagenesis retained only 19 amino acids of its tail. The portion of the *STE3* gene that was mutagenized included part of the third transmembrane segment and transmembrane segments 4 through 7, as well as the second and third extracellular loop (Fig. 2). Random mutagenized PCR fragments were mixed with a plasmid containing the *STE3* gene linearized by digestion with *Pme*I at a single site in the first part of the coding region. M18 cells transformed with the DNA mixture were screened on X‐Gal plates as we did for *STE2* (see Fig. 1A). Out of about 4000 screened colonies, we found 129 colonies displaying a white or light‐blue color. Plasmids were rescued and sequenced, yielding 29 candidates that had wild‐type *STE3* sequence, seven that had deletions, 42 with frameshift or stop‐codon mutations, and 58 that had missense mutations so distributed: 37 single mutants, 10 double mutants, three with three mutations, and one with four mutations. Fifty‐eight plasmids bearing missense mutations were further analyzed using quantitative *β*‐Gal assays of pheromone‐induced transcription (some typical mutants are shown in Fig. 1B).

Each of the mutations displaying an activity lower than 60% of the activity of the wild‐type Ste3 receptor were transferred into the full‐length receptor gene by PCR methods. Multiple mutations were separated from each other (see Table S1). The activity of the mutants was analyzed by a *β*‐Gal assay in the M18 strain and in DDS4, which is a strain isogenic to M18 except that it is wild‐type regarding *ITC1* and *SST2*, and therefore is neither supersensitive nor autocrine (Table S2). All the mutants in the second and third extracellular loop, as well as those in the second and third intracellular loop, were also analyzed in DDS2, which is supersensitive but not autocrine for the Ste3 receptor. Pheromone response activation in the nonautocrine strains was achieved using a cell‐free broth of a culture of *MAT* **a** cells as a source of **a**‐factor. As we did for the Ste2 receptor, we standardized the experimental conditions, making sure that *MAT* **a** cells used as a source of **a**‐factor were at the same level of time incubation and optical density in each experiment, repeating the assays several times. Moreover, in each assay, we compared mutant receptors against the wild‐type receptor, as an internal standard.

**Figure 5:**
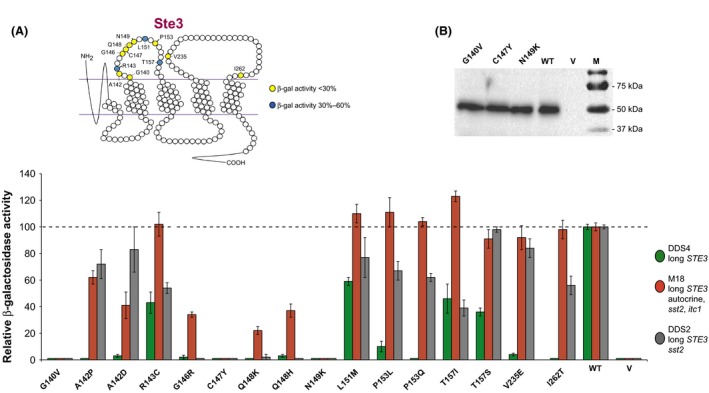
Analysis of selected mutations in the extracellular loops of the Ste3 receptor in different strains. (A) The position of mutated residues of the Ste3 receptor is depicted on the top. The *β*‐Gal activity of different strains carrying plasmids coding for the indicated mutants is shown. Data are normalized with respect to wild type. The horizontal line shows the value for the wild‐type receptor. The main features of the strains are indicated. The *itc1* mutation confers an autocrine character for the Ste3 receptor. (B) Western blot. The DDS4 strain was transformed with plasmids coding for the indicated mutant or wild‐type (WT) receptors, or with the empty vector (V). Total cell lysates (20 *μ*g each sample) were loaded on polyacrylamide gels and analyzed as indicated in the legend of Fig. 4.

Figure 5 shows the characterization of some mutations in the extracellular region or at the border with the transmembrane segments in full‐length receptors. For several residues, two different mutations were found, such as A142P/D, Q148K/H, P153L/Q, T157I/S. It is remarkable that the second extracellular loop is a sort of hot spot of mutations, with a change or even two in almost every position. In the corresponding loop of the Ste2 receptor, mutations were much fewer (Fig. 2).

For some mutants in the extracellular loops of the Ste3 receptor (R143C, L151M, P153L, P153Q, T157I), the activity was improved in the M18 strain (autocrine and *sst2*) up to a level comparable to the wild‐type one, indicating that the down‐phenotype originally displayed in the first screening (Fig. 1) was dependent on the absence of the C‐terminal region. In mutants R143C, L151M, and T157I, the activity in the *sst2* strain DDS2 is similar to that in the *SST2* ^+^ DDS4 strain but sensibly lower than in the autocrine M18 strain (Fig. 5). This finding might be explained by a lower affinity of the mutant receptors to the pheromone that is relieved in the autocrine strain, as for the Ste2 receptor. In other cases, the *sst2* trait of the DDS2 strain was sufficient to increase the down phenotype close to the wild‐type level (A142P and A142D, T157S, V235E).

For three residues (positions 140, 147, 149), neither the supersensitive nor the autocrine trait, nor the two together, suppressed the down mutation; therefore, these can be considered null mutants. For other mutants (positions 146 and 148), when the autocrine and the *sst2* traits are both present, the recovery is only partial. In order to ascertain that in the null mutants (G140V, C147Y and N149K) the receptor protein is being synthesized, a Western analysis was performed. Mutant Ste3 receptors are present at a level comparable to that of wild type, indicating that our null mutations do not affect the synthesis or the stability of the receptor (Fig. 5B). We also checked the expression of other null or down mutants by Western analysis. In all of them, there was no significant decrease in the amount of receptor expression.

**Figure 6:**
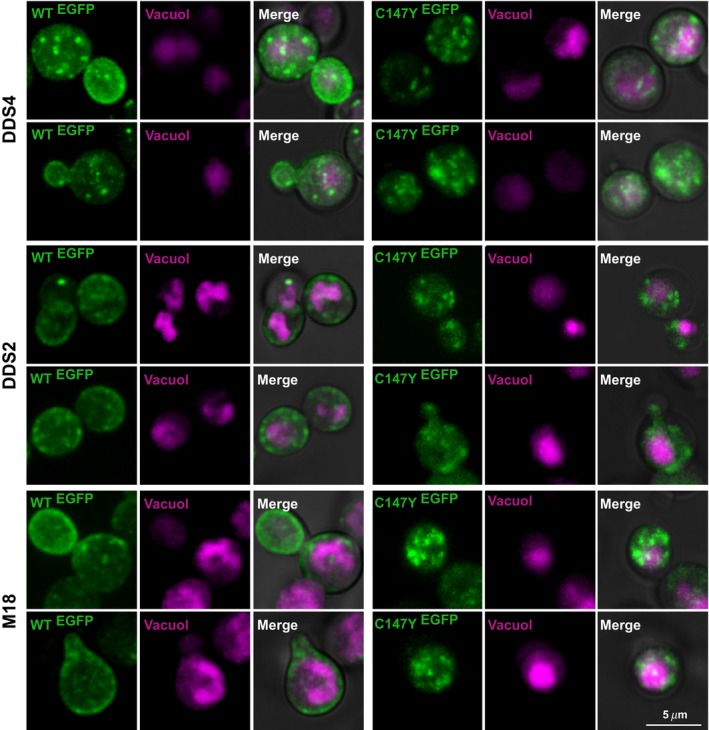
Confocal microscopy analysis on Ste3‐GFP (WT) and mutant Ste3‐C147Y‐GFP in DDS4, DDS2, and M18 strain, two samples each. Wild‐type Ste3‐GFP receptor (*green*) appears to be localized at the cell membrane and in vesicles around the vacuolar lumen (*magenta*). In C147Y mutant, GFP signal (*green*) is localized in vesicles throughout the cytoplasm and at the perivacuolar area (*magenta*). Not in all cells the vacuole was visible. GFP, green fluorescent protein.

Wild‐type and mutant receptors were observed furthermore by confocal microscopy using a receptor‐GFP fusion protein. Several strains were analyzed: DDS4, DDS2, and M18. Wild‐type Ste3‐GFP receptor molecules are located at the cell membrane and around the vacuolar lumen in all these strains. In the Ste3‐C147Y mutant, the GFP signal localizes throughout the cytoplasm and at the perivacuolar area, but apparently not at the cell membrane, in any of the strains that were tested (Fig. 6).

### *STE3* hyperactive mutants

**Figure 7:**
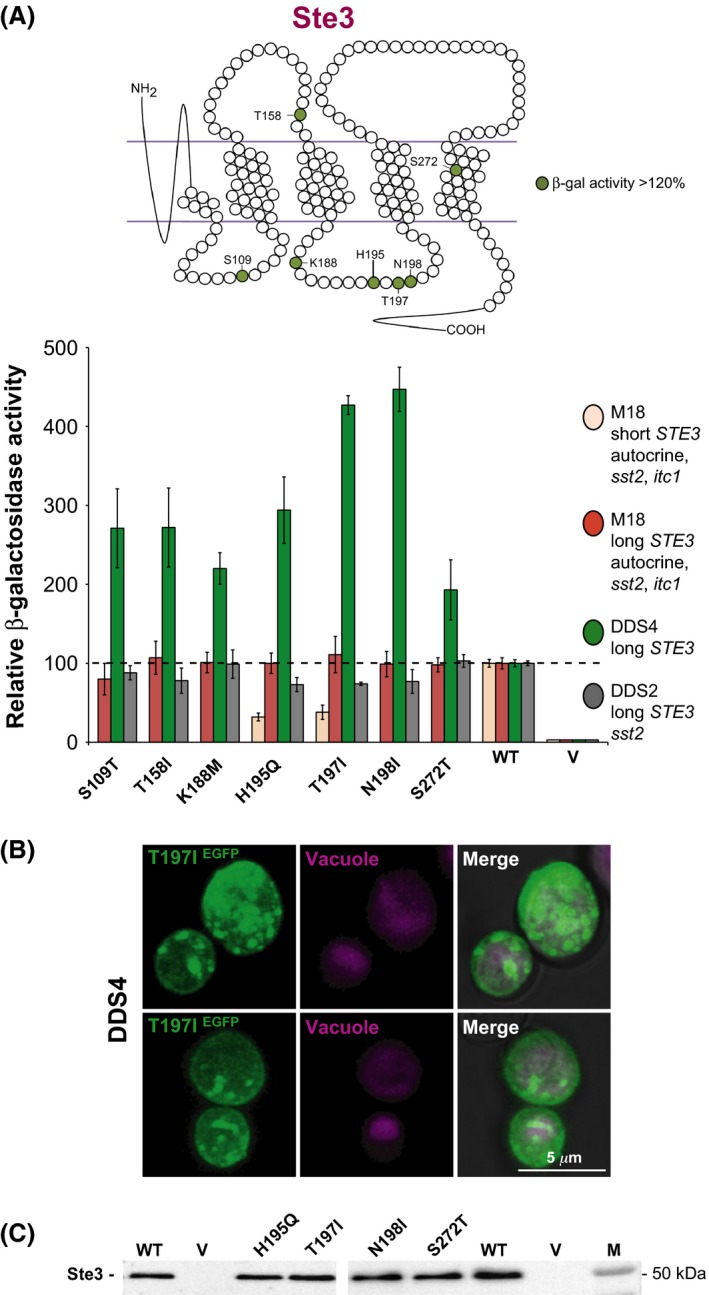
Hyperactive mutants of the Ste3 receptor. (A) The position of mutated residues with relative *β*‐Gal activity higher than the wild‐type Ste3 receptor is depicted on the top. The activity of different strains carrying plasmids coding for the indicated mutants is shown. Data are normalized with respect to wild type. The horizontal line shows the value for the wild‐type receptor. The main features of the strains are indicated. Autocrine M18 cells were incubated for 2.5 h in the absence of exogenous **a**‐factor: short and full‐length mutant receptors were analyzed, as indicated. DDS4 and DDS2 cells were incubated for 4 h together with a cell‐free broth of an overnight culture of *MAT* **a** strain GDS31, as a source of **a**‐factor. Hyperactivity is detected only in DDS4 cells. (B) Confocal microscopy analysis on Ste3‐GFP (WT) and hyperactive mutant Ste3‐T197I‐GFP in DDS4 strain. Mutant receptor (*green*) is highly expressed and localized at the cell membrane and throughout the cytoplasm. (C) Western blot. Total cell lysates (20 *μ*g each sample) of DDS4 cells transformed with plasmids coding for the indicated mutant or wild‐type (WT) receptors, or with the empty vector (V), were loaded on polyacrylamide gels and analyzed as indicated in the legend of Fig. 4. GFP, green fluorescent protein.

Among the *STE3* mutations, we found several mutants that displayed an interesting characteristic, that is, they caused a superactive phenotype, a feature not generally observed. Although the original screening of random mutants was meant to identify “down” mutations, we obtained eight *STE3* mutants that exhibit a higher activity in the mating‐response pathway compared to the wild‐type receptor. Two of the down mutants originally found harbored multiple mutations, and when we separated the amino acid changes from each other and analyzed them, we observed that some mutations strongly decreased the activity, but others caused an increase in the mating response. In such cases, the down mutations were clearly *cis*‐dominant with regard to the “up” mutations (Table S2). In one case, the double mutant was more hyperactive than the sum of the two single “up” mutations (Fig. S4). Among the “up” mutations found in the Ste3 receptor, one is localized in the second cytoplasmic loop, one at the border between the second intracellular loop and the fourth transmembrane segment, four in the third cytoplasmic loop, one in the second extracellular loop, and one in the last transmembrane segment (Fig. 7A). A mutation at the latter position, S272, is hyperactive when the codon change produces threonine, but when the mutation is to tyrosine or phenylalanine, the result is a receptor that is, respectively, null or highly defective (Table S2 and Fig. 7A).

Activities higher than wild type were observed only for mutants expressed in the DDS4 strain, which is neither supersensitive nor autocrine. Experiments were done under standardized conditions regarding time of incubation and optical density of cultures, and were repeated several times. Mutant receptors were compared, in the same experiment, to the wild‐type one, as an internal standard.

In strains that are autocrine or supersensitive, such as M18 and DDS2, the “up” mutants behave as wild type, or even as “down” mutants if analyzed in C‐terminus‐truncated receptors (see H195Q and T197I; Fig. 7A). The reason for this behavior is probably to be found in the extreme superactivity of the hyperactive mutants and truncated receptors in a strain that is also *sst2* and has the autocrine trait. All these features increase activity of the mating‐response pathway and it is conceivable that cell metabolism is affected when they are all present together (Stefan and Blumer 1994).

Confocal analysis showed that the hyperactive mutant Ste3‐T197I‐GFP, which is more than four times active with respect to the wild‐type Ste3 receptor, is highly expressed and localized at the cell membrane and throughout the cytoplasm (Fig. 7B).

To determine the level of expression of the Ste3 receptor in the hyperactive mutants, we did Western blot analysis. The “up” mutant receptors were expressed at levels similar to those of the wild‐type receptor, indicating that the hyperactive phenotype is not due to overexpression of the receptor (Fig. 7C for some of them).

Although in the screening described here, we found eight hyperactive mutations out of 58 mutants of *STE3*, we found none among 68 mutants of *STE2*. This difference appears to be statistically significant according to the *T*‐test (*P* = 0.007).

We wondered if hyperactive mutants of the Ste2 receptor could be obtained using a different approach. Since *STE2* with the endogenous promoter is scarcely expressed in the M18 strain, it is possible to screen for up mutants using X‐Gal plates that produce colonies with a deep blue color. Full‐length *STE2* DNA was mutagenized with the same PCR‐method used before, transferred into M18, and plated on X‐Gal plates. Ten blue colonies were found out of about 2000. Plasmids were rescued and sequenced. Two candidates harbored the N271I mutation, one candidate had a single change, R234S, and one candidate was mutated in two sites, K225N and A229T. Six candidates had wild‐type *STE2*. We inserted the three mutants into the plasmid pSM1, containing the *TPI* promoter, and tested them in RM6, which is, for the *STE2* gene, the analogous strain to DDS4 for *STE3*, where the “up” phenotype was observed. No hyperactivity was found for any of these *STE2* mutants in RM6 (Fig. S5).

## Discussion

The analysis of mutations in the yeast *STE2* and *STE3* genes can lead to a better understanding of the mechanisms that regulate signal transduction involving G protein‐coupled receptors.

In the present work, we detected dozens of different mutations in several regions of both receptors. Our approach was not to perform an extensive characterization of every mutant we detected, but instead, to compare the results obtained for these two receptors, which are different in their sequence but bind the same G protein, and draw inferences about their general nature.

A number of mutations found in this study were detected previously by other groups. Table 1 and 2 summarize the data for the Ste2 and Ste3 receptor, respectively, found in this work and in other studies. For example, mutation Y266C in the Ste2 receptor was shown to be strongly defective in the studies of Dosil et al. (1998), Leavitt et al. (1999) and Dube et al. (2000); our findings are consistent with their work. The activity of this mutant could be recovered in supersensitive strains (see Fig. 4), a result that was also obtained by Leavitt et al. (1999).

**Table 1:** Mutations found in the Ste2 receptor in this study. Mutations found in the same positions by other groups are also indicated

Amino acid	Mutation in this study	Mutation in other studies		Comments
M180	K (Fig. 4)	R	Dosil et al., 1998;	Involved in receptor conformation
M189	K (Fig. 3)			Activity rescue in autocrine cells
N194	S (Fig. 3)			Activity rescue in autocrine cells
F204	L (Fig. 4)	S	Dosil et al., 1998;	Involved in receptor conformation
N205	H (Fig. 4)	K	Dosil et al., 1998;	Involved in receptor conformation
		K–D	Lee et al., 2006	
		A	Naider et al., 2007	
S207	P (Fig. 4)	F	Dosil et al., 1998;	Involved in receptor conformation
L211	V (Fig. 4)			
S213	P (Fig. 4)			
S214	P (Fig. 4)			
S219	T (Fig. 3)			
V223	I (Fig. 3)			
R233	G (Fig. 3)	S	Weiner et al., 1993	R233G: Activity rescue in autocrine cellsDefect in receptor‐Gα coupling
		A	Clark et al., 1994;	
		C	Choi et al., 2006	
L236	I (Fig. 3)H–P (Fig. 4)	H–R	Weiner et al., 1993;	L236I: Activity rescue in autocrine cells Defect in receptor‐Gα coupling
		A	Clark et al., 1994;	
		C	Choi et al., 2006	
		F–W–H	Celic et al., 2003;	
K239	R (Fig. 3)	S	Celic et al., 2003;	Defect in receptor‐Gα coupling
		N	Strader et al., 1994;	
		A	Clark et al., 1994;	
		C	Choi et al., 2006	
F241	S (Fig. 3)	A	Clark et al., 1994;	Defect in receptor‐Gα coupling
		C	Choi et al., 2006	
H245	P (Fig. 3)	C	Choi et al., 2006	Defect in receptor‐Gα coupling
L248	P (Fig. 3)	C	Choi et al., 2006	L248P: Activity rescue in autocrine cellsL248C: Defect in receptor‐Gα coupling
C252	R (Fig. 4)	L	Dube et al., 1998	C252R: Strong defect; C252L: No defect
V257	D (Fig. 4)			
I261	K (Fig. 4)			
L264	H (Fig. 3)	P	Dosil et al., 1998;	L264H: Activity rescue in autocrine cellsL264P: Involved in receptor conformation
Y266	C (Fig. 4)	A–S–F–K–L	Lee et al., 2002	Involved in receptor conformation
		D–C	Dosil et al. 1998;	
		C	Dube et al.,^2000^	
		D–K	Lee et al., 2006	
		A	Naider et al., 2007	
N271	Y (Fig. 3)			
L277	V (Fig. 3)			
S288	F (Fig. 3)	P	Sommers et al., 2000;	Involved in receptor conformation
		A	Dube et al., 1998	

**Table 2:** Mutations found in the Ste3 receptor in this study (no mutations were found in the same positions by other groups)

Amino acid	Mutation in this study	Comments
S109	T (Fig. 7)	Hyperactive mutant
L117	H (Fig. S4)	Slightly hyperactive mutant
L121	M–F (Table S2)	
P124	L (Table S2)	
M126	R (Table S2)	
V127	I (Table S2)	
M128	R (Table S2)	
S131	P (Table S2)	
G140	V (Fig. 5)	
A142	P–D (Fig. 5)	Null mutant
R143	C (Fig. 5)	Activity rescue in autocrine cells
G146	R (Fig. 5)	Null mutant
C147	Y (Figs. 5, 6)	Null mutant
Q148	K–H (Fig. 5)	Null mutant
N149	K (Fig. 5)	Null mutant
L151	M (Fig. 5)	Activity rescue in autocrine cells
P153	L–Q (Fig. 5)	
T157	I–S (Fig. 5)	Activity rescue in autocrine cells
T158	I (Fig. 7)	Hyperactive mutant
T162	I (Table S2)	
M163	R (Table S2)	
I167	K (Table S2)	
W168	R (Table S2)	
S169	P (Table S2)	
K188	M (Fig. 7)	Hyperactive mutant
H195	Q (Fig. 7)	Hyperactive mutant
T197	I (Fig. 7)	Hyperactive mutant
N198	I (Fig. 7)	Hyperactive mutant
R208	M (Table S2)	
L209	P (Table S2)	
F212	I (Table S2)	
C213	Y–R (Table S2)	C213Y: Null mutantC213R: Down mutant
V235	E (Fig. 5)	Null mutant
I262	T (Fig. 5)	Null mutant
S272	T (Fig. 7)Y (Table S2)F (Table S2)	S272T: Hyperactive mutantS272Y: Null mutantS272F: Down mutant

Some mutant features are common in both the Ste2 and Ste3 receptor. For several cases, defective mutants recover their activity when they are assayed in an autocrine strain (see Fig. 3 for Ste2 and Fig. 5 for Ste3). A likely explanation of this finding is that the defect causes a lower affinity to the pheromone. This low affinity might be relieved in the autocrine strain if the local concentration of the pheromone is higher in the vicinity of the receptor, due to the receptor being physically close to the area where the pheromone is secreted (Brizzio et al. 1996).

It is remarkable that the second extracellular loop of Ste3 is a sort of hot spot of mutations, with a change or even two in almost every position. In the corresponding loop of the Ste2 receptor, mutations were much fewer (Fig. 2). The loss‐of‐function mutations in this region of Ste3 are essentially of two types: those that are null or highly defective in every strain in which they were assayed, and those where the activity is recovered, in some cases up to the wild‐type level, when expressed in an autocrine and supersensitive strain. One mutation, T158I, is even hyperactive (see below).

The confocal microscopy observation showed a different behavior of the Ste2 and Ste3 receptors. Both receptors fused to the GFP localized at the cell membrane or within and around the vacuolar lumen when they were analyzed in nonautocrine strains. However, in autocrine cells, the GFP signal diffused throughout the cytoplasm in cells expressing the Ste2 receptor, while for the Ste3 receptor, it localized at the cell membrane. It appears, therefore, that autocrine cells expressing the Ste3 receptor behave differently from those expressing Ste2. Although for wild‐type Ste3, a continuous activation such as that occurring in an autocrine strain is tolerated, this is not the case for Ste2. Consistently, null mutants of both receptors localized at the perivacuolar area in nonautocrine as well as in autocrine strains.

The most interesting result of our study is the detection of several hyperactive mutations in the Ste3 receptor. Many diseases are associated with malfunction of GPCRs, either because of loss of function or because of gain of function (reviewed in Schöneberg et al. 2004; Vassart and Costagliola 2011). The gain‐of‐function mutations detected in the Ste3 receptor induce an activity in signal transduction that is up to fivefold over the level of wild‐type Ste3.

One of the “up” mutations of the Ste3 receptor is remarkable. In position S272, when the codon change produces tyrosine or phenylalanine, the result is a receptor that is, respectively, null or highly defective. On the other hand, if the change is to the threonine codon, this mutant is hyperactive at about 200% level (Table S2 and Fig. 7A). Both tyrosine and phenylalanine carry an aromatic ring, while serine and threonine do not. Since position 272 is located in the seventh transmembrane segment, it is possible that the aromatic ring causes a defect in the structure of the receptor or affects the binding to the pheromone. This is the only case where we found so strong a difference in mutations affecting the same residue.

Some of the “up” mutations of the Ste3 receptor are located in the third intracellular loop, a result that was also observed by Boone et al. (1993). One hyperactive mutation was found in the second intracellular loop, suggesting that it is also implicated in the binding of the Gα subunit.

In one case, a double mutant was more hyperactive than the sum of the two single “up” mutations (Fig. S4). One of the two residues is located in the middle of the second intracellular loop, and the other one is placed at the junction between this loop and the fourth transmembrane segment. It is possible that both residues are involved in the binding of the Gα subunit or that they interact with each other producing a more efficient receptor molecule.

The main difference between the Ste2 and Ste3 receptor that we observed in this work was that while hyperactive mutations were found for Ste3, we did not observe any gain‐of‐function mutant for the Ste2 receptor. We interpret this finding as an outcome of the different strategies the two genes have adopted for their expression (Tsong et al. 2003; Galgoczy et al. 2004; Tsong et al. 2006; Li and Johnson 2010). In the regulatory circuit of the yeast mating system, the *STE2* gene must be expressed in *MAT* **a** cells but repressed in *MAT*α cells, and conversely in the case of *STE3*. For this kind of regulation to be effective, the repression of *STE2* in *MAT*α cells should be very rigorous. In fact, if *MAT*α cells, due to leakiness in the system, produced even a small amount of the opposite receptor, that is, Ste2, they would be autocrinely activated by the α‐factor that they secrete. The activation of the mating pathway, in turn, would lead to growth arrest and therefore to lethality.

In view of this consideration, it is easy to understand that since the *STE2* gene is negatively regulated and must always be tightly repressed in *MAT*α cells, any hyperactive mutation would be harmful. In fact, if some synthesis of the Ste2 receptor occurred as a result of loss of repression, the higher the performance of the Ste2 protein in signal transduction, the worse the consequence will be, since it will cause a stronger autocrine response. It is therefore plausible that the Ste2 polypeptide has reached the almost maximum possible level of function as a receptor and that no hyperactive mutations are conceivable.

On the other hand, for the *STE3* gene, which is positively regulated and is expressed only in *MAT*α cells, hyperactive mutations will be better tolerated even in the other cell type, *MAT* **a**, since the probability that the *STE3* gene is transcribed in *MAT* **a** cells is very low.

Support for this interpretation is found in our previous work, where we found that the *STE2* and *STE3* genes are differently regulated at the transcriptional and the polyadenylation level (Di Segni et al. 2011). In that study, we showed that a cryptic polyadenylation site is present inside the early coding region of the *STE2* gene, but no such site is found in the *STE3* gene, where only the regular poly(A) site in the 3′ untranslated end is present. The two cell types, *MAT* **a** and *MAT*α, produce an incomplete *STE2* transcript, but only *MAT* **a** cells generate a full‐length *STE2* mRNA.

Several previous studies, especially that of Shah and Marsh (1996), reported that signaling responses for the Ste2 receptor are essentially independent of levels of *STE2* expression. This means that even low amounts of the Ste2 receptor, such as those derived by escaping the repression of transcription, could have harmful effects.

The *STE2* and *STE3* genes appear to be very different, not only at the level of their DNA and the derived amino acid sequences but also regarding their transcriptional and post‐transcriptional regulation. Here, we have shown that they are also distant from each other regarding the characteristics of the proteins encoded by them. These receptors are a good example of convergent evolution, shedding new insight on signal transduction and receptor function.

## Conflict of Interest

None declared.

## Supporting information


**Figure S1.** Western blot.

**Figure S2.** Quantitative *β*‐Gal assay of wild‐type Ste2 receptor fused to the GFP or not, espresse in the M18 strain.

**Figure S3.** Confocal microscopy analysis on Ste2‐GFP (WT) and mutant Ste2‐S207P‐GFP in DDS4, DDS2 and M18 strains.

**Figure S4.** Activity of a double‐mutant Ste3 receptor is higher than that of each single mutant. The position of the two mutations of Ste3 is indicated.

**Figure S5.** Analysis of mutations in the Ste2 receptor.

**Table S1.** Primers for the subcloning of mutations from C‐truncated forms (short) of the *STE2* and *STE3* genes to full‐length (long) *TPI‐STE2* and *TPI‐STE3*.

**Table S2.** Mating response induced by C‐truncated (short) and full‐length (long) forms of single and multiple *STE2* and *STE3* mutants, respectively, in RM6 and DDS4 strain.

**Table S3.** List of the oligodeoxyribonucleotide primers used for sequencing and PCR.
