# Supplementary material for: Analysis of random PCR‐originated mutants of the yeast Ste2 and Ste3 receptors
Source: Microbiologyopen. 2016 May 5;5(4):670–86. doi: 10.1002/mbo3.361 (PMC4985600; doi:10.1002/mbo3.361)
Supplement: Supplementary file 1 — Figure S1. Western blot. [file MBO3-5-670-s001.pdf]

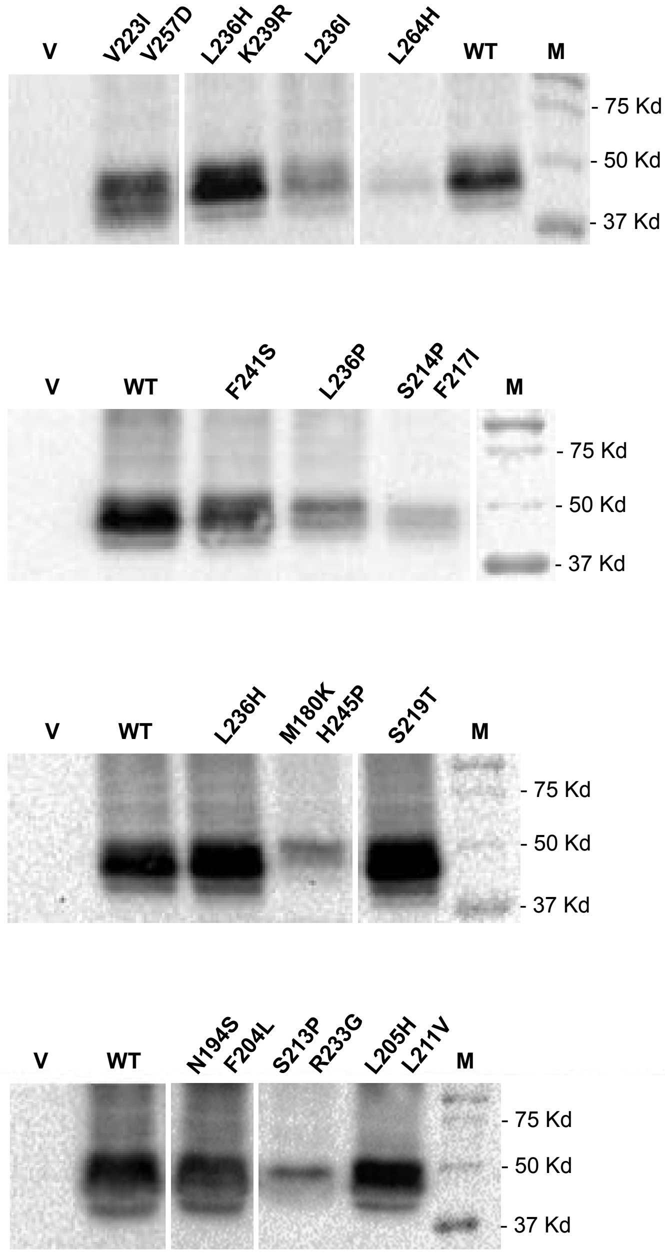

Figure S1. Western blot. The RM6 strain was transformed with plasmids coding for the indicated mutant or wild-type (WT) receptors, or with the empty vector (V). Total cell lysates (30 µg each sample) were loaded on polyacrylamide gels and analyzed by western blot using anti-HA antibodies. Protein standards are shown in lane M.
