# Supplementary material for: Analysis of random PCR‐originated mutants of the yeast Ste2 and Ste3 receptors
Source: Microbiologyopen. 2016 May 5;5(4):670–86. doi: 10.1002/mbo3.361 (PMC4985600; doi:10.1002/mbo3.361)
Supplement: Supplementary file 2 — Figure S2. Quantitative β‐Gal assay of wild‐type Ste2 receptor fused to the GFP or not, espresse in the M18 strain. [file MBO3-5-670-s002.pdf]

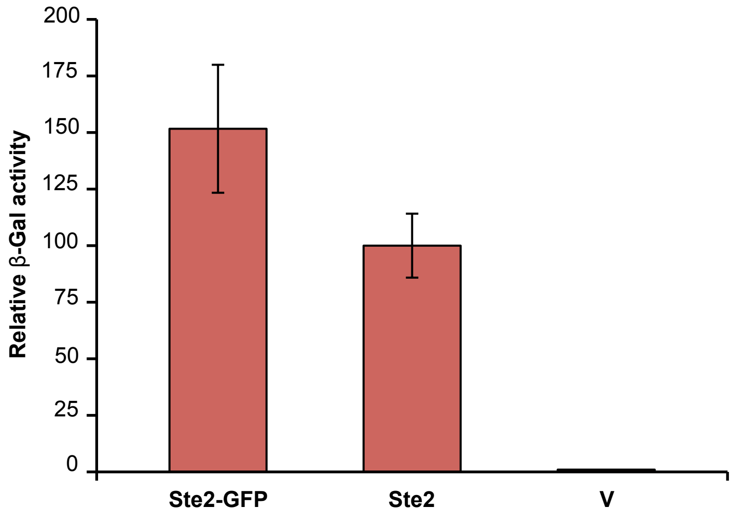

Figure S2. Quantitative β-Gal assay of wild-type Ste2 receptor fused to the GFP or not, expressed in the M18 strain. The results represent the averages of three samples, normalized to the level of β-Gal activity of cells expressing the wild-type Ste2 receptor; error bars correspond to 1 standard deviation (SD).
