# Supplementary material for: Analysis of random PCR‐originated mutants of the yeast Ste2 and Ste3 receptors
Source: Microbiologyopen. 2016 May 5;5(4):670–86. doi: 10.1002/mbo3.361 (PMC4985600; doi:10.1002/mbo3.361)
Supplement: Supplementary file 3 — Figure S3. Confocal microscopy analysis on Ste2‐GFP (WT) and mutant Ste2‐S207P‐GFP in DDS4, DDS2 and M18 strains. [file MBO3-5-670-s003.pdf]

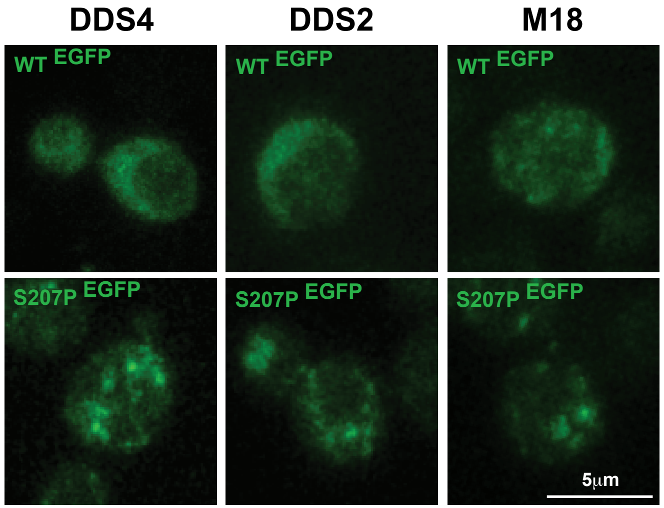

Figure S3. Confocal microscopy analysis on Ste2-GFP (WT) and mutant Ste2-S207P-GFP in DDS4, DDS2 and M18 strains. In these strains the vacuole was not visible
