# Supplementary material for: Analysis of random PCR‐originated mutants of the yeast Ste2 and Ste3 receptors
Source: Microbiologyopen. 2016 May 5;5(4):670–86. doi: 10.1002/mbo3.361 (PMC4985600; doi:10.1002/mbo3.361)
Supplement: Supplementary file 4 — Figure S4. Activity of a double‐mutant Ste3 receptor is higher than that of each single mutant. The position of the two mutations of Ste3 is indicated. [file MBO3-5-670-s004.pdf]

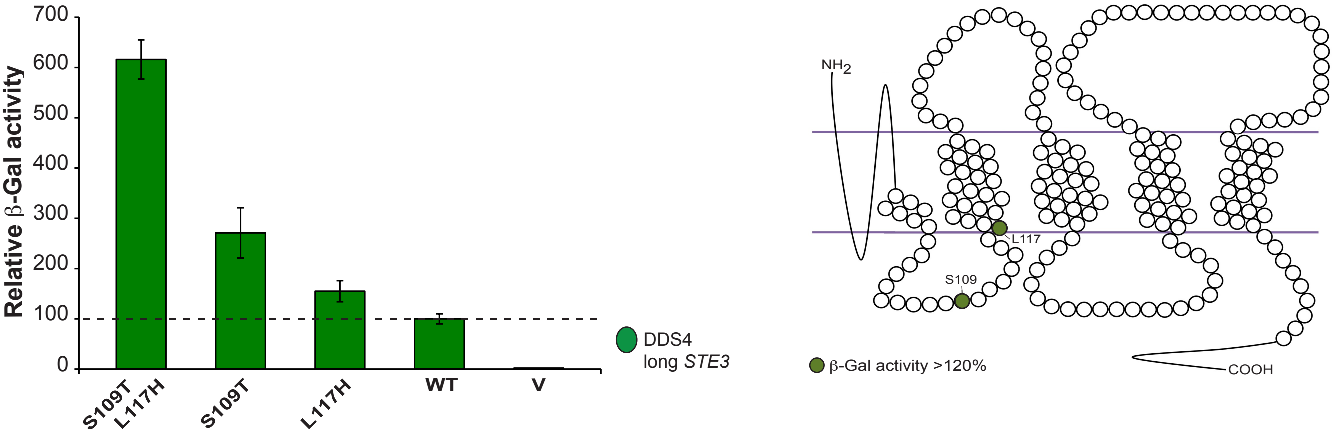

Figure S4. Activity of a double mutant Ste3 receptor is higher than that of each single mutant. The position of the two mutations of Ste3 is indicated. The DDS4 strain was transformed with a plasmid carrying the double mutant gene, the two single mutant genes, or the wild-type receptor. The empty vector is indicated by V. The relative β-Gal activity of permeabilized cells is shown. The horizontal line indicates the value for wild-type receptor.
