# Supplementary material for: Analysis of random PCR‐originated mutants of the yeast Ste2 and Ste3 receptors
Source: Microbiologyopen. 2016 May 5;5(4):670–86. doi: 10.1002/mbo3.361 (PMC4985600; doi:10.1002/mbo3.361)
Supplement: Supplementary file 5 — Figure S5. Analysis of mutations in the Ste2 receptor. [file MBO3-5-670-s005.pdf]

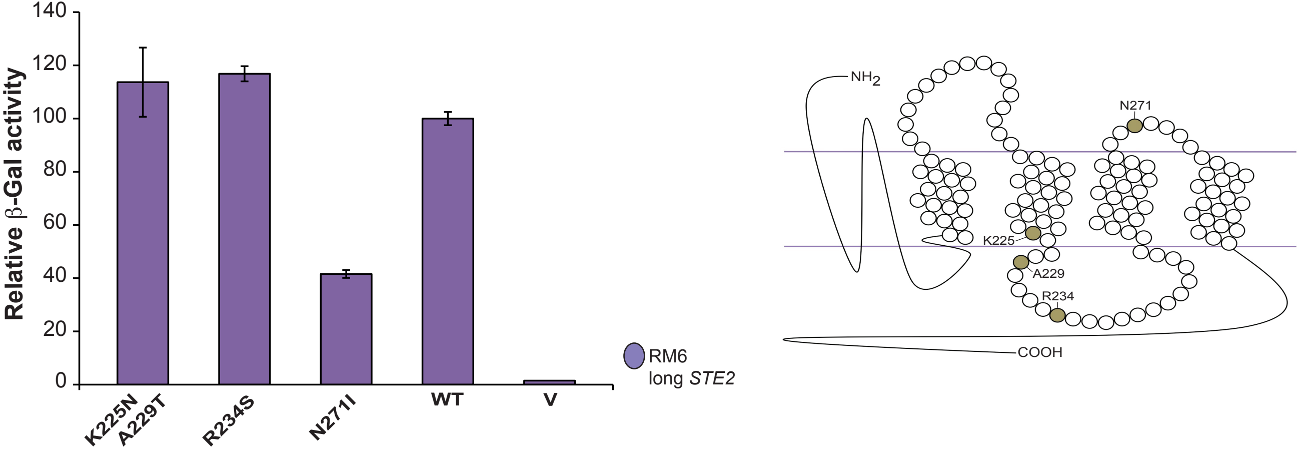

Figure S5. Analysis of mutations in the Ste2 receptor. The position of mutated residues is indicated in the schematic representation of the receptor. Normalized β-Gal activity of RM6 strain carrying the Ste2 mutant genes is shown.
