# Supplementary material for: Analysis of random PCR‐originated mutants of the yeast Ste2 and Ste3 receptors
Source: Microbiologyopen. 2016 May 5;5(4):670–86. doi: 10.1002/mbo3.361 (PMC4985600; doi:10.1002/mbo3.361)
Supplement: Supplementary file 6 — Table S1. Primers for the subcloning of mutations from C‐truncated forms (short) of the STE2 and STE3 genes to full‐length (long) TPI‐STE2 and TPI‐STE3. [file MBO3-5-670-s006.doc]

**Table S1.** Primers for the subclonig of mutations from C-truncated forms (short) of the *STE2* and *STE3* genes to full-lenght (long)

*TPI-STE2* and *TPI-STE3*.

| ***TPI-STE2* SINGLE MUTATION** | **FIRST PCR** | **SECOND PCR** |
| --- | --- | --- |
| S207P - S219T - K225R  L228S - L236H - L236I  L236P - K239M - F241S  S243N - L247F - L248P - C252R | short *STE2* mutant: P8-P6 |  |
| L264H - Y266C - L277V | short *STE2* mutant: P9-P10 |  |
| P258Q - S259L - I261K | short *STE2* mutant: P8-P6 pSM1: P40-P10 | P8-P10 |
| ***TPI-STE2* DOUBLE MUTATION** | **FIRST PCR** | **SECOND PCR** |
| M180K  H245P | short *STE2* mutant: P8-P22 pSM1: P21-P10 | P8-P10 |
|  | short *STE2* mutant: P21-P10 pSM1: P8-P22 | P8-P10 |
| M189K  S214P | short *STE2* mutant: P8-P16 pSM1: P9-P6 | P8-P6 |
|  | short *STE2* mutant: P9-P6 pSM1: P8-P16 | P8-P6 |
| N194S  F204L | short *STE2* mutant: P8-P18 pSM1: P17-P6 | P8-P6 |
|  | short *STE2* mutant: P17-P6 pSM1: P8-P18 | P8-P10 |
| V196A  S219L | short *STE2* mutant: P8-P16 pSM1: P9-P10 | P8-P10 |
|  | short *STE2* mutant: P9-P10 pSM1: P8-P16 | P8-P10 |
| N205H  L211V | pSM1: P8-P31 pSM1: P32-P10 | P8-P10 |
|  | pSM1: P8-P33 pSM1: P34-P10 | P8-P10 |
| S213P  R233G | short *STE2* mutant: P8-P20 pSM1: P19-P6 | P8-P6 |
|  | short *STE2* mutant: P19-P6 pSM1: P8-P20 | P8-P6 |
| S214P  F217I | pSM1: P8-P23 pSM1: P24-P10 | P8-P10 |
|  | pSM1: P8-P25 pSM1: P26-P10 | P8-P10 |
| V223I  V257D | short *STE2* mutant: P8-P22 pSM1: P21-P10 | P8-P10 |
|  | short *STE2* mutant: P21-P10 pSM1: P8-P22 | P8-P10 |
| L236H  K239R | pSM1: P8-P27 pSM1: P28-P10 | P8-P10 |
|  | pSM1: P8-P29 pSM1: P43-P10 | P8-P10 |
| I260L  L287R | pSM1: P8-P42 pSM1: P43-P10 | P8-P10 |
|  | pSM1: P8-P44 pSM1: P45-P10 | P8-P10 |
| L264H  N271Y | pSM1: P8-P35 pSM1: P36-P10 | P8-P10 |
|  | pSM1: P8-P37 pSM1: P38-P10 | P8-P10 |
| S267R  S288F | short *STE2* mutant: P8-P41 pSM1: P39-P10 | P8-P10 |
|  | short *STE2* mutant: P39-P10 pSM1: P8-P41 | P8-P10 |

| ***TPI-STE3* SINGLE MUTATION** | **FIRST PCR** | **SECOND PCR** |
| --- | --- | --- |
| M126R - M128R - G129E - A142D  R143C - G146R - C147Y - Q148H  P153L - T157I - M163R - H195Q - T197I | short *STE3* mutant: P11-P3 |  |
| P124L | short *STE3* mutant: P11-P12 |  |
| E208M - V225D - V235E - Δ(L201-L203) | short *STE3* mutant: P11-P3 pSM3: P14-P15 | P11-P15 |
| C213R - C213Y - S272F | short *STE3* mutant: P13-P3 pSM3: P14-P15 | P13-P15 |
| L209P - N149K - I262T - S272Y | selected on long TPI *STE3* |  |
| ***TPI-STE3* DOUBLE MUTATION** | **FIRST PCR** | **SECOND PCR** |
| S109T  L117H | pSM3: P11-P71 pSM3: P70-P73 | P11-P73 |
|  | pSM3: P11-P79 pSM3: P78-P73 | P11-P73 |
| L121M  S131P | long *STE3* mutant: P11-P54 pSM3: P55-P73 | P11-P73 |
|  | long *STE3* mutant: P55-P73 pSM3: P11-P54 | P11-P73 |
| L121F  G140V  K188M | short *STE3* mutant: P11-P54 pSM3: P55-P73 | P11-P73 |
|  | long *STE3* mutant: P72-P73 pSM3: P11-P54 | P11-P73 |
|  | long *STE3* mutant: P72-P73 pSM3: P11-P72 | P11-P73 |
| V127I  A142P | pSM3: P11-P73 pSM3: P72-P73 | P11-P73 |
|  | pSM3: P11-P65 pSM3: P64-P73 | P11-P73 |
| Q148K  T162I | pSM3: P11-P59 pSM3: P58-P73 | P11-P73 |
|  | short *STE3* mutant: P72-P73 pSM3: P11-P53 | P11-P73 |
| L151M  W168R | short *STE3* mutant: P11-P69 pSM3: P68-P73 | P11-P73 |
|  | short *STE3* mutant: P72-P73 pSM3: P11-P53 | P11-P73 |
| P153Q  T157I  F212I | pSM3: P11-P67 pSM3: P66-P73 | P11-P73 |
|  | N.A. |  |
|  | long *STE3* mutant: P72-P3 pSM3: P14-P15 pSM3: P11-P53 | P11-P15 |
| T157S  A176P  K186E  S272T | long *STE3* mutant: P11-P69 pSM3: P68-P73 | P11-P73 |
|  | pSM3: P11-P75 pSM3: P74-P73 | P11-P73 |
|  | pSM3: P11-P57 pSM3: P56-P73 | P11-P73 |
|  | long *STE3* mutant: P72-P15 pSM3: P11-P73 | P11-P15 |
| T158I  G172S | pSM3: P11-P77 pSM3: P76-P73 | P11-P73 |
|  | long *STE3* mutant: P68-P73 pSM3: P11-P69 | P8-P10 |
| T162I  V171M | N.A. |  |
|  | long *STE3* mutant: P68-P73 pSM3: P11-P69 | P11-P73 |
| I167K  S169P | pSM3: P11-P61 pSM3: P60-P73 | P11-P73 |
|  | pSM3: P11-P63 pSM3: P62-P73 | P11-P73 |
| L150S  N191I | N.A. |  |
|  | long *STE3* mutant: P72-P73 pSM3: P11-P53 | P11-P73 |
