# Supplementary material for: Analysis of random PCR‐originated mutants of the yeast Ste2 and Ste3 receptors
Source: Microbiologyopen. 2016 May 5;5(4):670–86. doi: 10.1002/mbo3.361 (PMC4985600; doi:10.1002/mbo3.361)
Supplement: Supplementary file 7 — Table S2. Mating response induced by C‐truncated (short) and full‐length (long) forms of single and multiple STE2 and STE3 mutants, respectively, in RM6 and DDS4 strain. [file MBO3-5-670-s007.doc]

**Table S2.** Mating response induced by C-truncated (short) and full-length (long) forms of single and multiple *STE2* and *STE3* mutants, respectively in RM6 and DDS4 strain. Values are represented as the mean of 3 samples ± 1 standard deviation relative to the wild-type gene. Column 2 and 5 show the relative β-Gal activity induced by C-truncated mutant receptors under autocrine activation in the supersensitive strain M18, while column 3 and 6 show the relative activity of full-length mutated receptors in RM6 or DDS4 strain that are neither autocrine nor supersensitive. Column 7 shows the relative activity of single full-length mutants for each multiple mutant in RM6 or DDS4 strain. N. D. = Not Determined.

| ***STE2***  **SINGLE**  **MUTATION** | **β-Gal ACTIVITY (%WT)** | | ***STE2***  **MULTIPLE**  **MUTATION** | **β-Gal ACTIVITY (%WT)** | | |
| --- | --- | --- | --- | --- | --- | --- |
|  | **M18**  **SHORT Ste2** | **RM6**  **LONG Ste2** |  | **M18 SHORT Ste2**  **MULTIPLE MUTANT** | **RM6 LONG Ste2**  **MULTIPLE MUTANT** | **RM6 LONG Ste2**  **SINGLE MUTANT** |
| S207P | 9 ± 6 | 1,3 ± 0,8 | M180K / H245P | 10 ± 4 | 6 ± 3 | 2 ± 0,5 / 47 ± 8 |
| S219T | 25 ± 10 | 47 ± 4 | M189K / S214P | 0 | 4 ± 2 | 7 ± 4 / 0 |
| K225R | 30 ± 4 | 120 ± 23 | N194S / F204L | 47 ± 5 | 0 | 0 / 2 ± 1 |
| L228S | 13 ± 2 | 97 ± 6 | V196A / S219L | 60 ± 0,4 | 66 ± 14 | 92 ± 19 / 106 ± 6 |
| L236H | 30 ± 11 | 3 ± 0,8 | N205H / L211V | 0 | 0 | 0 / 21 ± 4 |
| L236I | 15 ± 1 | 10 ± 3 | S213P / R233G | 5 ± 4 | 0 | 6 ± 3 / 6 ± 2 |
| L236P | 0 | 5 ± 3 | S214P / F217I | 25 ± 9 | 5 ± 2 | 0 / 116 ± 35 |
| K239M | 24 ± 4 | 82 ± 18 | V223I / V257D | 18 ± 10 | 7 ± 1 | 56 ± 11 / 10 ± 3 |
| F241S | 27 ± 3 | 46 ± 8 | L236H / K239R | 3,4 ± 1,5 | 16 ± 5 | 0 / 55 ± 9 |
| S243N | 14 ± 3 | 75 ± 20 | I260L / L287R | 41 ± 4 | 130 ± 27 | 17 ± 11 / 120 ± 28 |
| L247F | 21 ± 6 | 112 ± 20 | L264H / N271Y | 18 ± 6 | 22 ± 4 | 38 ± 4 / 47 ± 10 |
| L248P | 16 ± 1 | 9 ± 2 | S267R / S288F | 0 | 107 ± 7 | 84 ± 15 / 53 ± 5 |
| C252R | 5 ± 0,3 | 0 |  |  |  |  |
| S259L | 56 ± 7 | 109 ± 5 |  |  |  |  |
| I261K | 42 ± 1 | 0 |  |  |  |  |
| L264H | 22 ± 4 | 67 ± 5 |  |  |  |  |
| Y266C | 27 ± 3 | 0 |  |  |  |  |
| L277V | 11 ± 4 | 45 ± 11 |  |  |  |  |

| ***STE3***  **SINGLE**  **MUTATION** | **β-Gal ACTIVITY (%WT)** | | ***STE3***  **MULTIPLE**  **MUTATION** | **β-Gal ACTIVITY (%WT)** | | | | |
| --- | --- | --- | --- | --- | --- | --- | --- | --- |
|  | **M18**  **SHORT Ste3** | **DDS4**  **LONG Ste3** |  | **M18 SHORT Ste3**  **MULTIPLE MUTANT** | | **DDS4 LONG Ste3**  **MULTIPLE MUTANT** | | **DDS4 LONG Ste3**  **SINGLE MUTANT** |
| P124L | 36 ± 4 | 34 ± 2 | S109T / L117H | 42 ± 5 | | 616 ± 39 | | 271 ± 50 / 155 ± 21 |
| M126R | 0 | 24 ± 2 | L121M / S131P | 48 ± 3 | | 53 ± 10 | | 113 ± 8 / 53 ± 6 |
| M128R | 0 | 28 ± 3 | V127I / A142P | N.D. | | 2 ± 1 | | 24 ± 3 / 0 |
| G129E | 43 ± 9 | 114 ± 8 | Q148K / T162I | 15 ± 4 | | 0 | | 0 / 26 ± 5 |
| A142D | 38 ± 3 | 3 ± 1 | L151M / W168R | 47 ± 10 | | 0 | | 59 ± 3 / 5 ± 4 |
| R143C | 44 ± 21 | 43 ± 8 | T158I / G172S | 6 ± 11 | | 171 ± 55 | | 273 ± 50 / 32 ± 7 |
| G146R | 19 ± 8 | 2 ± 3 | T162I / V171M | 6 ± 1 | | 17 ± 5 | | 26 ± 5 / 65 ± 3 |
| C147Y | 0 | 0 | I167K / S169P | 0 | | 0 | | 52 ± 12 / 7 ± 6 |
| Q148H | 57 ± 4 | 3 ± 1 | L150S / N198I | N.D. | | 190 ± 2 | | N.D. / 447 ± 28 |
| N149K | N.D. | 0 | L121F / G140V / K188M | 0 | | 0 | | 77 ± 5 / 0 /  220 ± 20 |
| P153L | 61 ± 4 | 10 ± 4 |  |  |  |  |  |  |
| T157I | 45 ± 20 | 46 ± 11 | P153Q / T157I / F212I | | 0 | | 0 | 0 / 46 ± 11 /  38 ± 6 |
| M163R | 66 ± 6 | 47 ± 3 |  |  |  |  |  |  |
| H195Q | 32 ± 5 | 294 ± 42 | T157S / A176P / K186E / S272T | | N.D. | | 1 ± 1 | 36 ± 3 / 0 /  109 ± 3 /193 ± 38 |
| T197I | 38 ± 9 | 427 ± 12 |  |  |  |  |  |  |
| Δ(L201-L203) | 15 ± 1 | 67 ± 16 |  | |  | |  |  |
| R208M | 29 ± 10 | 54 ± 3 |  | |  | |  |  |
| L209P | 29 ± 8 | 40 ± 4 |  |  | |  | |  |
| C213R | 0 | 1 ± 1 |  |  | |  | |  |
| C213Y | 1 ± 1 | 8 ± 2 |  |  | |  | |  |
| V225D | 11 ± 5 | 83 ± 3 |  |  | |  | |  |
| V235E | 32 ± 5 | 4 ± 1 |  |  | |  | |  |
| I262T | N.D. | 0 |  |  | |  | |  |
| S272Y | N.D. | 1 ± 1 |  |  | |  | |  |
| S272F | 7 ± 3 | 8 ± 4 |  |  | |  | |  |
