# Supplementary material for: Analysis of random PCR‐originated mutants of the yeast Ste2 and Ste3 receptors
Source: Microbiologyopen. 2016 May 5;5(4):670–86. doi: 10.1002/mbo3.361 (PMC4985600; doi:10.1002/mbo3.361)
Supplement: Supplementary file 8 — Table S3. List of the oligodeoxyribonucleotide primers used for sequencing and PCR. [file MBO3-5-670-s008.doc]

**Table S3.** List of the oligodeoxyribonucleotide primers used for sequencing and PCR.

| **P1** 5’-TATCCGTATGATGTGCCTGACTACGCATGATCAAAATTTACGGCTTTG-3’ | **P41** 5’-CAGCAAGTAATGTTGCAACAGTAGTC-3’ |
| --- | --- |
| **P2** 5’-TGCGTAGTCAGGCACATCATACGGATATAAATTATTATTATCTTCAGTCC-3’ | **P42** 5’-AGGATGAATATGATCGATGGAACCAAC-3’ |
| **P3** 5’-AAGTACAAATCCTAGTTTGATGGA-3’ | **P43** 5’-GTTGGTTCCATCGATCATATTCATCCT-3’ |
| **P4** 5’-CATATCCAAGAATTCCTATCAAAATTTTCATGTCATACAAGTCAG-3’ | **P44** 5’-AATGGTAAAGACTTTACAGCAAGTAATG-3’ |
| **P5** 5’-TCACTTTAGGGATTGCTACAGTTACCATGTA-3’ | **P45** 5’-CATTACTTGCTGTAAAGTCTTTACCATT-3’ |
| **P6** 5’-GCCCACATTGATGATAATGGT-3’ | **P46** 5’-GCTAAAATCAAATTAACTACCAGG-3’ |
| **P7** 5’-ACAACTTGCATACAATTTTGAAGGCA-3’ | **P47** 5’-CCTGGTAGTTAATTTGATTTTAGC-3’ |
| **P8** 5’-CATATCCAAGAATTCCCAATCAAAAATGTCT-3’ | **P48** 5’-CTTCTTGATCTAATAGTTAAAATCAAT-3’ |
| **P9** 5’-TACTTCAATGCATCCACAATTTTACTTGCA-3’ | **P49** 5’-ATTGATTTTAACTATTAGATCAAGAAG-3’ |
| **P10** 5’-CGGTCAGTCATTAATACTAGGAACAAGAGA-3’ | **P50** 5’-CCCAAGCTTAAAGGAGAAGAACTTTTCACTGG-3’ |
| **P11** 5’-ATGTCATACAAGTCAGCAATAATA-3’ | **P51** 5’-CCGCTCGAGCTATTTGTATAGTTCATCCATGCC-3’ |
| **P12** 5’-TTATGGGTAAGGTCGTGGTAGACCTT-3’ | **P52** 5’-AAAACTGCAGGCCCTAAAGGAGAAGAACTTTTCACTGG-3’ |
| **P13** 5’-aacttattgtctccgacatggattacc-3’ | **P53** 5’-GGTAATCCATGTCGGAGACAATAAGTTTTGG-3’ |
| **P14** 5’-CCTGCGTTCCATCAAACTAGGATTTGTA-3’ | **P54** 5’-TCCCATGACCATGACAGGTGTGAA-3’ |
| **P15** 5’-TTTTCTAGGATCATCTGGAATTCCTATAGT-3’ | **P55** 5’-TTCACACCTGTCATGGTCATGGGA-3’ |
| **P16** 5’-TGCAAGTAAAATTGTGGATG-3’ | **P56** 5’-CGTGTTTTATAAAGAACGCAAGGACG-3’ |
| **P17** 5’-ATGTTAGTGCCACCCAAGAT-3’ | **P57** 5’-CGTCCTTGCGTTCTTTATAAAACACG-3’ |
| **P18** 5’-ATCTTGGGTGGCACTAACAT-3’ | **P58** 5’-CGTTACAACGGTTGCAAAAACTTATTGTCTCCG-3’ |
| **P19** 5’-ATGTCATTTGTCCTGGTAGTTAAA-3’ | **P59** 5’-CGGAGACAATAAGTTTTTGCAACCGTTGTAACG-3’ |
| **P20** 5’-TTTAACTACCAGGACAAATGACAT-3’ | **P60** 5’-tgtggatgcttaaatggtcatttgt-3’ |
| **P21** 5’-GATCAAGAAGATTCCTTGGTCTCAAGC-3’ | **P61** 5’-acaaatgaccatttaagcatccaca-3’ |
| **P22** 5’-GCTTGAGACCAAGGAATCTTCTTGATC-3’ | **P62** 5’-atgcttatatggccatttgtgggcg-3’ |
| **P23** 5’-GACATAAAGTTTATTGGGGATGCAAG-3’ | **P63** 5’-cgcccacaaatggccatataagcat-3’ |
| **P24** 5’-CTTGCATCCCCAATAAACTTTATGTC-3’ | **P64** 5’-tttagatatggtatccctcgttacaacgg-3’ |
| **P25** 5’-TGACATAATGTTTATTGAGGATGCAAG-3’ | **P65** 5’-ccgttgtaacgagggataccatatctaaa-3’ |
| **P26** 5’-CTTGCATCCTCAATAAACATTATGTCA-3’ | **P66** 5’-aaaacttattgtctcagacatggattaccac-3’ |
| **P27** 5’-CGAACTGCTTGAGACCATGGAATC-3’ | **P67** 5’-gtggtaatccatgtctgagacaataagtttt-3’ |
| **P28** 5’-GATTCCATGGTCTCAAGCAGTTCG-3’ | **P68** 5’-GTTTTGTATACCATGTGGATGCTTATATGG-3’ |
| **P29** 5’-TCGAACTGCCTGAGACCAAGGAATC-3’ | **P69** 5’-CCATATAAGCATCCACATGGTATACAAAAC-3’ |
| **P30** 5’-GATTCCTTGGTCTCAGGCAGTTCGA-3’ | **P70** 5’-CCGGATCTTTCAACATGGACGAAAATCGTC-3’ |
| **P31** 5’-ATGCAAGTAAAATTGTGGATGCATGGAAGTA-3’ | **P71** 5’-GACGATTTTCGTCCATGTTGAAAGATCCGG-3’ |
| **P32** 5’-TACTTCCATGCATCCACAATTTTACTTGCAT-3’ | **P72** 5’-GTTCACACCTGTCATGATCATGGGATTTTC-3’ |
| **P33** 5’-ATGCAACTAAAATTGTGGATGCATTGAAGTA-3’ | **P73** 5’-GAAAATCCCATGATCATGACAGGTGTGAAC-3’ |
| **P34** 5’-TACTTCAATGCATCCACAATTTTAGTTGCAT-3’ | **P74** 5’-GGGCGCTGTTTATCCCACCTTAGTACTGTTC-3’ |
| **P35** 5’-CTGGTTTGGTTTCAAACTGTATGCGTGGAT-3’ | **P75** 5’-GAACAGTACTAAGGTGGGATAAACAGCGCCC-3’ |
| **P36** 5’-ATCCACGCATACAGTTTGAAACCAAACCAG-3’ | **P76** 5’-CCGACATGGATTACCATTGTTTTGTATACC-3’ |
| **P37** 5’-CTGGTATGGTTTCAAACTGTATGCGAGGAT-3’ | **P77** 5’-GGTATACAAAACAATGGTAATCCATGTCGG-3’ |
| **P38** 5’-ATCCTCGCATACAGTTTGAAACCATACCAG-3’ | **P78** 5’-TCGTCAAGGACCATGTGATTAGCTTGTTC-3’ |
| **P39** 5’-GACTACTGTTGCAACATTACTTGCTG-3’ | **P79** 5’-GAACAAGCTAATCACATGGTCCTTGACGA-3’ |
| **P40** 5’-ACCATTATCATCAATGTGGGCC-3’ |  |
